# The Relationship between Mating System and Genetic Diversity in Diploid Sexual Populations of *Cyrtomium falcatum* in Japan

**DOI:** 10.1371/journal.pone.0163683

**Published:** 2016-10-05

**Authors:** Ryosuke Imai, Yoshiaki Tsuda, Sadamu Matsumoto, Atsushi Ebihara, Yasuyuki Watano

**Affiliations:** 1 Department of Biology, Graduate School of Science, Chiba University, Yayoi, Inage, Chiba, Japan; 2 Sugadaira Montane Research Center, University of Tsukuba, Ueda, Nagano, Japan; 3 Department of Botany, National Museum of Nature and Science, Tsukuba, Ibaraki, Japan; National Cheng Kung University, TAIWAN

## Abstract

The impact of variation in mating system on genetic diversity is a well-debated topic in evolutionary biology. The diploid sexual race of *Cyrtomium falcatum* (Japanese holly fern) shows mating system variation, i.e., it displays two different types of sexual expression (gametangia formation) in gametophytes: mixed (M) type and separate (S) type. We examined whether there is variation in the selfing rate among populations of this species, and evaluated the relationship between mating system, genetic diversity and effective population size using microsatellites. In this study, we developed eight new microsatellite markers and evaluated genetic diversity and structure of seven populations (four M-type and three S-type). Past effective population sizes (Ne) were inferred using Approximate Bayesian computation (ABC). The values of fixation index (*F*_IS_), allelic richness (*A*_*R*_) and gene diversity (*h*) differed significantly between the M-type (*F*_IS_: 0.626, *A*_*R*_: 1.999, *h*: 0.152) and the S-type (*F*_IS_: 0.208, *A*_*R*_: 2.718, *h*: 0.367) populations (when admixed individuals were removed from two populations). Although evidence of past bottleneck events was detected in all populations by ABC, the current *N*_e_ of the M-type populations was about a third of that of the S-type populations. These results suggest that the M-type populations have experienced more frequent bottlenecks, which could be related to their higher colonization ability via gametophytic selfing. Although high population differentiation among populations was detected (*F*_ST_ = 0.581, *F’*_ST_ = 0.739), there was no clear genetic differentiation between the M- and S-types. Instead, significant isolation by distance was detected among all populations. These results suggest that mating system variation in this species is generated by the selection for single spore colonization during local extinction and recolonization events and there is no genetic structure due to mating system.

## Introduction

The diversity of mating and sexual systems in land plants is of great interest to evolutionary biologists. For seed plants, the presence of both male and female reproductive organs within a single individual (i.e. monoecy) is a common and ancestral state [1]. Thus, seed plants are potentially faced with a strategic decision of whether to reproduce through outcrossing, selfing, or mixed mating, which is a mixture of outcrossing and selfing [2]. In the short term, selfing is favored due to transmission advantage [3] and reproductive assurance [4,5]. However the progeny derived from selfing may suffer inbreeding depression [6]. The relative balance between these advantages and disadvantages strongly influences mating system evolution. In this respect, the correlation between the evolution of selfing and severe genetic drift events such as bottlenecks is important because both the transmission advantage [3] and reproductive assurance [4,5] theories predict their co-occurrence. The former theory predicts that population bottleneck events that result in the purging of inbreeding depression would trigger the evolution of selfing [7], while in the latter theory, selfing would be favored under limited mating opportunities, which are expected in colonization processes. Thus, if populations display mating system variation, reduction of effective population size is expected, due to the co-occurrence of selfing and bottlenecks [8]. However, this scenario has not been well examined in either gymnosperm or pteridophyte species. The mating systems of homosporous pteridophytes are of interest because they are unique among vascular plants; their free-living haploid gametophytes can bear both male and female gametangia (antheridia and archegonia) and they are capable of three types of mating systems: gametophytic selfing (i.e. syngamy of gametes derived from the same gametophyte; this extreme form of inbreeding is not possible in heterosporous plants), sporophytic selfing (i.e. syngamy of gametes from two gametophytes that developed from separate spores from the same parent sporophyte; this is analogous to selfing in seed plants), and outcrossing (i.e. syngamy of gametes from two gametophytes derived from separate sporophytes) [9,10]. In order to deepen our understanding of the evolution of mating systems in plants, it is important to examine the correlation between mating system and genetic diversity in homosporous pteridophytes, which are uniquely capable of extreme inbreeding via gametophytic selfing.

**Fig 1:**
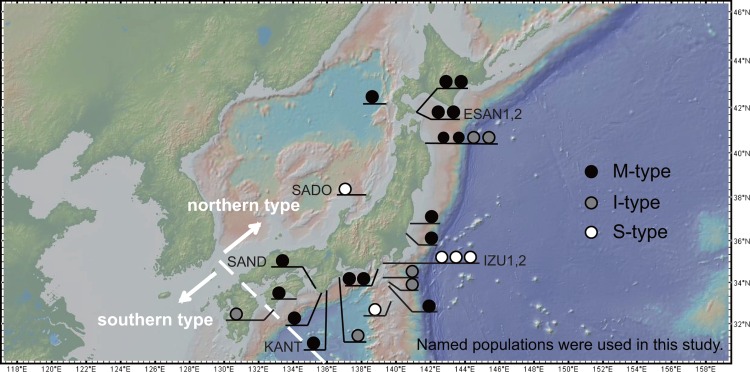
Distribution of northern and southern types of diploid sexual *Cyrtomiun falcatum* in Japan based on Matsumoto (2003) [11], and sampling locations of 7 populations examined in this study. Dashed line is the boundary of the northern and southern type’s distributional range. This map was drawn by GeoMapApp (http://www.geomapapp.org/). Locations of the M-type (black circle), the I-type (gray circle) and the S-type (open circle) sporophyte individuals identified by Matsumoto (2003) [11] are shown.

In this study, we focused on Japanese holly fern, *Cyrtomium falcatum* (L.f.) C.Presl (Dryopteridaceae), to evaluate the relationship among sexual expression of gametophytes, mating system and genetic diversity. Matsumoto (2003) [11] divided the sexual diploid cytotype of *Cyrtomium falcatum* in Japan into northern and southern types. The northern type is distinguished from the southern type by smaller blades, fewer pairs of pinnae, and grayish indusia without a blackish brown center [11]. The northern type grows on coastal rocks or cliffs and is distributed discontinuously from the southern part of Hokkaido Island to Shikoku Island [11], while the southern type is distributed across the Okinawa Islands, Kyusyu Island and Ogasawara Islands (Fig 1). Matsumoto (2003) [11] observed variation in the sexual expression of the northern type of *C*. *falcatum*. Sporophytes called mixed type (M-type) simultaneously produce gametophytes with both antheridia (male gametangia) and archegonia (female gametangia) at frequencies of 90% or greater after three months in cultivation. In contrast, sporophytes of the separate type (S-type) produce these bisexual gametophytes at frequencies of 10% or less. This sexual expression is equivalent to dichogamy, in which an individual temporally staggers the development of gametangia, because the gametophytes produce antheridia in the early stages of development and then produce archegonia after releasing sperm. Sporophytes that produce intermediate frequencies (10% < frequency < 90%) of gametophytes that produce antheridia and archegonia simultaneously are considered to be the intermediate type (I-type). Matsumoto (2003) [11] confirmed that all southern type individuals were the S-type. Traditionally, the S-type of gametangia formation on gametophytes (female at maturity) has been considered a morphological adaptation to promote outcrossing and the M-type (simultaneous hermaphrodite at maturity) enables gametophytic selfing [9,12]. In support of this hypothesis, gametophytes of the M-type sporophyte can produce sporophytes at very high rates (84%–100%) in isolated cultures, whereas the S-type sporophytes show a low rate of sporophyte formation (5%–35%) [11]. Ranker and Houston (2002) [13] compared the sexual expression of natural gametophytes with that of cultured gametophytes for the Hawaiian fern *Sadleria*, and suggested that the occurrence of simultaneously hermaphroditic (considered to be equivalent to the M-type) and unisexual gametophytes (S-type) in the laboratory would also be a good predictor of their occurrence in nature. Therefore, the S- and M-types observed in *C*. *falcatum* could be considered to reflect mating system variation in nature. Understanding this morphological variation is important because it helps clarify the role of mating systems in shaping genetic diversity of populations and may provide clues to the causes of mating system evolution. In the present study, we developed eight new microsatellite markers to evaluate genetic variation within and among populations of the northern type of *C*. *falcatum*. The specific aims of the present study were to: 1) test whether the wild populations of M-type individuals show higher selfing rates than those of the S-type individuals, 2) test correlations between high selfing rates, low genetic diversity and reduction of effective population size, if the selfing rate varies among populations, 3) estimate the past and current effective population size, and 4) test whether the M-type and the S-type populations are genetically differentiated.

## Materials and Methods

### Populations and sampling

The gametangia formation types, M-type or S-type, of the seven *C*. *falcatum* populations we examined are as determined by Matsumoto (2003) [11]. Briefly, Matsumoto (2003) [11] sowed spores on agar medium, and then transplanted each of the obtained gametophytes separately (e.g. one gametophyte in one separate well) onto vermiculite medium in the laboratory. At three months after spore sowing, forty gametophytes per sporophyte were examined to evaluate gametangia formation types [11]. Because each gametophyte was cultivated in isolation, variance in sexual expression by density [14] could be ignored. In Matsumoto’s (2003) [11] experiment, 33 sporophyte individuals of the northern type of diploid *C*. *falcatum* from 20 localities (Fig 1) were examined.

For this study, we collected 233 sporophyte samples from seven populations (21–42 individuals per population) in five localities of sexual diploid populations of *C*. *falcatum* that were previously examined by Matsumoto (2003) [11]. The seven populations and their localities were as follows: ESAN 1 (41.8112N, 141.1844E) and ESAN 2 (41.8115N, 141.1844E) from Esan-misaki, Hokkaido Prefecture; SADO (38.0929N, 138.2498E) from Sado Island, Niigata Prefecture; IZU 1 (34.8824N, 139.1323E) and IZU 2 (34.8821N, 139.1319E) from Jogasaki, Shizuoka Prefecture; KANT (33.6004N, 135.6004E) from Kantori-misaki, Wakayama Prefecture; and SAND (33.6655N, 135.3355E) from Sandan-peki, Wakayama Prefecture (Fig 1). Three populations (IZU 1, IZU 2, SADO) were selected from the localities where the S-types have been observed, and four populations (ESAN 1, ESAN 2, SAND, KANT) were collected from the localities where M-types have been observed [11].

Voucher specimens of the samples (ri010001–ri010233) were deposited in the Herbarium of the National Museum of Nature and Science (TNS), Tsukuba, Ibaraki, Japan. For sampling in Esan Prefectural Natural Park, we obtained permission from the Hokkaido Government Biodiversity Division.

### DNA extraction and microsatellite marker development

Total DNA was extracted from silica-dried leaves using the HEPES/CTAB method [15]. The DNA samples were used for microsatellite marker development and further genotyping. We developed microsatellite markers using two different methods; one was an improved technique for isolating co-dominant compound microsatellite markers [16] and the other was a next-generation sequencing (NGS) method [17].

Firstly, following the method of Lian *et al*. (2006) [16] genomic DNA of a sample from IZU1 was digested with six blunt-end cutters (*Hae*III, *Pvu*II, *Alu*I, *Ssp*I, *Eco*RI, and *Sca*I) and ligated with a specific blunt adaptor [18] using a T4 DNA Ligation kit (Nippon Gene, Tokyo, Japan). The digested and ligated fragments were amplified using a compound SSR primer (AC)_5_(AG)_8_ and an adaptor primer AP2 (5′-CTATAGGGCACGCGTGGT-3′) [16]. The PCR products were cloned using the TOPO-TA Cloning Kit (Invitrogen, Carlsbad, USA). Plasmid DNAs were amplified from the colonies with a TemliPhi DNA Amplification Kit (GE Healthcare Bio-Sciences, Little Chalfont, UK). Sequence reactions were prepared with T3 and T7 primers (Invitrogen) using the BigDye Terminator v3.1 Cycle Sequencing Kit (Applied Biosystems, Tokyo, Japan). The reaction mixture was analyzed on an ABI 3500 genetic analyzer (Applied Biosystems). A total of 576 different fragments with a compound microsatellite motif at one end were obtained. From 86 different sequences, specific primers were designed and selected to have 200 to 400bp product length using PRIMER3 software [19]. A PIG1-tail (5’-GTTTCTT-3’) was added to specific forward primers to reduce stuttering [20].

Secondly, we developed microsatellite markers using an NGS method with a Roche 454 Genome Sequencer Junior (Roche/454 Life Sciences, Branford, CT, USA). Genomic DNA was isolated from a pinna of the southern type of diploid *Cyrtomium falcatum*, collected from the Tsukuba Botanical Gardens (originally collected from Wakayama Pref., Japan) and fragmented by nebulization. A DNA library was constructed using the GS FLX Titanium Rapid Library Preparation Kit (Roche/454 Life Sciences). The DNA library was purified using the MinElute PCR Purification Kit (Qiagen, Tokyo, Japan) and its quality was checked using the Agilent High Sensitivity DNA kit (Agilent Technologies, Palo Alto, CA, USA). Emulsion PCR was carried out using the GS Junior Titanium emPCR Lib-L Kit (Roche/454 Life Sciences), and pyrosequencing was conducted on a Roche 454 Genome Sequencer Junior instrument at the Tsukuba Botanical Gardens, with the GS Junior Titanium Sequencing Kit (Roche/454 Life Sciences). Contigs were assembled to over 500bp with GS Newbler De Novo Assembler (Roche/454 Life Sciences), implementing the default parameters and heterozygotic mode. The program QDD v.2.1 [21] was used with default settings to detect and select microsatellite sequences. Twelve hundred contigs were used for searching microsatellite candidates. We designed 72 primer pairs based on the penalty scores calculated with Primer3 in the QDD pipeline. As described by Schuelke (2000) [22], the U19 sequence (5′-GGTTTTCCCAGTCACGACG-3′) was added to the 5′ end of specific forward primer sequences. A PIG2 tail (5’-GTTT-3’) was added to specific reverse primer sequences [20].

### Fragment analysis of microsatellite markers

We tested all of the candidate markers (86 by the method of Lian *et al*. (2006) [16] and 72 by the NGS method) for good PCR amplification, reproducibility, and the level of polymorphism over all samples, using a subset of samples: two individuals from each of the seven populations. Finally, eight primer pairs were selected and used to further genotype all samples (S1 Table). PCR amplifications (simplex PCR) were performed using the Multiplex PCR Kit (Qiagen) in a downscaled final volume of 5 μl according to the manufacturer's protocol. The forward and reverse primers were adjusted to 0.2 μM in final concentration and 20 ng of DNA was added to each reaction. For two primer sets, CFL-079 and CFL–C32, PCRs were conducted using each specific primer and a dye-labeled (AC)_6_(AG)_10_ primer (ABI PRISM®, Applied Biosystems) under the following conditions: initial denaturation for 15 min at 95°C, followed by 30 cycles at 95°C for 30 s, 55°C for 90 s, 72°C for 1 min, and final extension at 60°C for 30 min. The PCR reaction mixture for the other six primer sets contained 0.2 μM reverse primer, 0.04 μM forward primer, and 0.2 μM of the fluorescent dye-labeled U19 primer (ABI PRISM®, Applied Biosystems), which acted as the second forward primer for the cycles following the touchdown stage. Touchdown PCR was performed with initial denaturation for 15 min at 95°C, followed by 25 cycles at 95°C for 30 s, 63–53°C (with a 0.5°C decrease for every subsequent cycle) for 90 s, and 72°C for 1 min, followed by 20 cycles of 95°C for 30 s, 53°C for 90 s, and 72°C for 1 min, and final extension at 60°C for 30 min. The PCR products were analyzed on an ABI 3500 Genetic Analyzer (Applied Biosystems) with the internal size standard, GeneScan 600 LIZ (Applied Biosystems), and fragment sizes were determined with GeneMapper 3.1 (Applied Biosystems). The original sequences for the markers were deposited in GenBank under the accession numbers LC055975—LC055982 (S1 Table).

### Data analyses

#### Inbreeding coefficient and genetic diversity within populations

Gene diversity (*h*; [23]) and allelic richness (*A*_R_; [24]) were calculated for each population using FSTAT ver. 2.9.3.2 [25]. FSTAT was also used to test genotypic disequilibrium among loci for each population. We used INEST2 [26], with the ‘nfb’ model, to estimate *F*_IS_ values within populations, taking into account the effect of underestimating heterozygosity in the presence of null alleles. As mentioned previously, two types of self-fertilization may occur in homosporous ferns: gametophytic (*S*_I_) and sporophytic (*S*) selfing. Hedrick (1987) [27] derived a formula showing the relationship among *F*_IS_ and the rates of two types of selfing (*S*_I_ and *S*).

$$F_{IS}=\frac{S+2S_{I}}{2-S}$$

In the present study, we use *F*_IS_ values as an indicator of the relative contributions of selfing *sensu-lato* (both S and S_I_) to outcrossing [28]. To assess whether population genetic parameters differ between M- and S-type populations, inbreeding coefficients (*F*_IS_), gene diversity (*h*), allelic richness (*A*_R_), relatedness [29], and *F*_ST_ [30] values were calculated and compared, treating M- and S-types as two groups. Differences in these values between the two types were tested for significance using a randomization test in FSTAT. We employed one-sided *P*-values to test whether the value in one group is significantly larger than the other. Our STRUCTURE analysis (see below) indicated that cluster 6 at *K* = 6 was admixed in both SAND and SADO populations, although these two populations are located far apart from each other (Fig 1, see Results and Discussion for details). This cluster 6 was further divided into two clusters in *K* = 7 corresponding to each of the two populations. As we found that these admixed clusters affected the evaluation of genetic diversity in these two populations, we also analyzed genetic diversity in the two populations after removing individuals which had ancestry values of greater than 50% to cluster 6 in *K* = 6.

#### Genetic differentiation and structure among populations

Genetic differentiation among populations was evaluated by calculating the overall and pairwise *F*_ST_ [30] values and their respective confidence intervals (95%) were determined on the basis of 1000 bootstrapping replicates using FSTAT. The standardized values of *F*_ST_ and *F*’_ST_ [31] were also calculated using GenAlEx 6.5 [32]. Patterns of isolation by distance (IBD; [33]) were evaluated, using GenAlEx [32], according to the method described by Rousset (1997) [34]; a Mantel test (with 999 random permutations) between the matrices obtained for pairwise population differentiation in terms of *F*_ST_ /(1—*F*_ST_) and the natural logarithms of direct minimum geographic distance among populations. Genetic structure was also investigated with the model-based clustering algorithm implemented in the software STRUCTURE v. 2.3.3 [35,36]. A number of clusters (*K*) varying from 1 to 15, were evaluated under the correlated allele frequencies model by running 100,000 burn-in Markov Chain Monte Carlo (MCMC) repetitions and 1,000,000 subsequent repetitions based on the LOCPRIOR model [36]. The probabilities of each *K* were averaged over 10 runs. We employed the CLUMPAK server [37] to evaluate multimodality [38] among runs at each *K*. The optimum *K* value was determined based on *ΔK* [39], evaluating the probability of the data (Ln P(D)) for each *K* value using STRUCTURE HARVESTER [40]. Bar charts representing the proportion of cluster membership in each individual were obtained using CLUMPAK. The genetic relationships between the clusters were evaluated based on genetic distance calculated in STRUCTURE and a neighbor-joining tree of clusters was generated using Populations 1.2.23 [41].

#### Inference of past population size change and effective population size

The software DIYABC v2.0 [42,43] was used to infer past population size changes and the effective population size of *Cyrtomium falcatum* based on the Approximate Bayesian Computation (ABC) approach. DIYABC provides flexibility for the mutation models of microsatellite loci in coalescent simulations, allowing both the generalized stepwise mutation model (GSM; [44]) and the single nucleotide indel model (SNI). As our main purpose was to test whether the effective population size and demographic history among M- and S-type populations were different due to their different forms of gametangia formation, three simple scenarios were examined in each population (S1 Fig):

Scenario 1. Bottleneck model: the ancestral effective population size (*N*_a_) was changed at *t*_1_ to the modern effective population size (*N*_1_) and *N*_1_ was set to be smaller than *N*_a_.

Scenario 2. Constant model: the ancestral effective population size (*N*_b_) and the modern one (*N*_1_) were set to be equal, assuming the effective population size has not changed.

Scenario 3. Expansion model: the ancestral effective population size (*N*_c_) was changed at *t*_1_ to the modern effective population size (*N*_1_) and *N*_1_ was set to be larger than *N*_c_.

In these scenarios, *t*_1_ represents time scale measured by generation time. We employed the default values of the priors for each parameter in DIYABC. The mean values for expected heterozygosity (*H*_E_), number of alleles (*A*), allele size variance across loci and M index across loci [45,46] were used as summary statistics. A million simulations were run for each scenario. After all the simulations had been run, the most-likely scenario was determined by comparing the posterior probabilities using the logistic regression method. The goodness of fit of the scenario was assessed by the option ‘‘model checking” with principal component analysis (PCA) in DIYABC, which measures the discrepancy between the model and real data. To translate the inferred number of generations for *t*_1_ to time scale by year, we assumed a generation time of 3 years, as Matsumoto (2003) [11] showed that the northern type of *C*. *falcatum* produces spores at 1 year in cultivation tests.

## Results

### Characteristics of the eight microsatellite loci

Eight new microsatellite markers were developed in this study (S1 Table). The number of detected alleles ranged from 2 (locus CFL-B16) to 14 (locus CFL-B-02 and CFL-079) and a total of 64 alleles were detected across all 8 loci (S2 Table). The null allele frequencies estimated by INEST2 (S3 Table) were relatively high (over 0.10) in 9 out of 56 (8 loci × 7 populations) combinations, but significant in only one case: CFL-B12 locus in SADO (0.246; 95% CI: 0.0977–0.400). No significant deviations from genotypic equilibrium were detected once putative admixed individuals in SADO were excluded.

### Mating system and genetic diversity

All of the populations, except for SADO, showed significantly positive inbreeding coefficient (*F*_IS_) values, ranging from 0.220 to 0.794 over all loci (Table 1). The average *F*_IS_ value of M-type populations (0.626) was significantly higher than S-type (0.208, *P* < 0.05; Table 2). The *F*_IS_ values estimated using INEST2 (S4 Table) did not largely differ from those of FSTAT, suggesting that the presence of null alleles had little influence on the overall results. The average allelic richness (*A*_R_) of the M-type populations (1.999) was significantly lower than that of the S-type populations (2.718, *P* < 0.05; Table 2). Similarly, the average value of gene diversity (*h*) of the M-type populations (0.152) was significantly lower than that of the S-type populations (0.367, *P* < 0.05; Table 2). When admixed individuals were included in these analyses, different trends were not detected (Table 2).

**Table 1:** Genetic diversity indices and inbreeding coefficient values for seven populations of the northern type of diploid *Cyrtomium falcatum*.

	M-type populations				S-type populations		
	ESAN1	ESAN2	SAND	KANT	IZU1	IZU2	SADO
*N*_A_	2.130	2.125	2.500 (3.750)	2.250	3.750	2.625	3.000 (4.125)
*A*_R_	1.700	1.847	2.380 (3.626)	2.074	2.900	2.390	2.863 (3.776)
*h*	0.076	0.158	0.227 (0.374)	0.217	0.378	0.326	0.436 (0.562)
*F*_IS_	**0.501**	**0.671**	**0.794 (0.602)**	**0.560**	**0.340**	**0.220**	-0.100 **(0.157)**
*N*_1_	169	210	299	328	846	465	715

*N*_A_, mean number of alleles; *A*_R_, allelic richness; *h*, gene diversity; *F*_IS_, multilocus estimate of inbreeding coefficient; *N*_1_, current effective population size estimated by DIYABC. Bold type indicates significant *F*_IS_ values (p < 0.00089, simple Bonferroni correction for 5% level). Genetic diversity indices and inbreeding coefficient values in parentheses are those calculated for all samples including the admixed individuals revealed by the STRUCTURE analysis.

**Table 2:** Group comparison of population genetic parameters between the M- and the S-type populations of the northern type of diploid *Cyrtomium falcatum*.

	*A*_R_	*h*	*F*_IS_	Relatedness	*F*_ST_
M-type	1.999	0.152	0.626	0.708	0.663
(all samples)	(2.378)	(0.177)	(0.587)	(0.672)	(0.619)
S-type	2.718	0.367	0.208	0.490	0.367
(all samples)	(3.110)	(0.410)	(0.241)	(0.440)	(0.328)
*P*-value (M>S)	0.989	1.000	0.029*	0.064	0.067
(all samples)	(0.890)	(0.977)	(0.002*)	(0.560)	(0.270)
*P*-value (S>M)	0.016*	0.009*	0.981	0.963	0.967
(all samples)	(0.168)	(0.053)	(1.000)	(0.954)	(1.000)

*A*_R_, allelic richness; *h*, gene diversity; *F*_IS_, multilocus estimate of inbreeding coefficient; *F*_ST_, pairwise *F*_ST_ value among populations

*, *P*-values less than 0.05. Population genetic parameters calculated using all samples including the admixed individuals revealed by the STRUCTURE analysis are shown in parentheses.

### Population genetic structure

**Fig 2:**
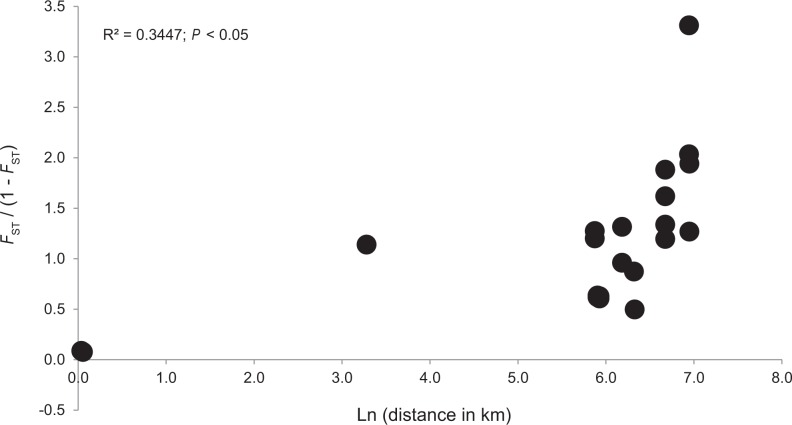
Isolation by distance for the 7 populations of the northern type of diploid *Cyrtomium falcatum*. The relationship between the matrix of pairwise differentiation described as *F*_ST_ / (1 − *F*_ST_) and the matrix of the natural logarithm of geographic distance (in meters) among the 7 populations.

**Fig 3:**
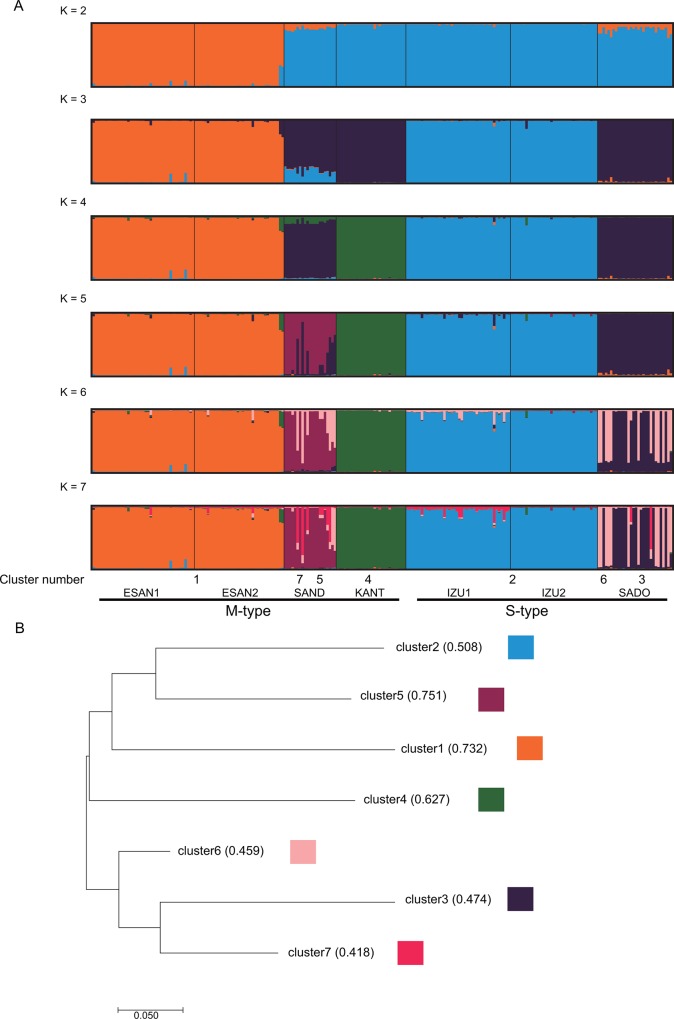
Results of the STRUCTURE analysis and the Neighbor-joining (NJ) tree of the clusters when *K* = 7. (A) The proportion of the membership coefficient of 233 individuals in the 7 populations for each of the inferred clusters for *K* = 2–7 defined using Bayesian clustering in STRUCTURE analysis. Population types and cluster numbers on the NJ tree are indicated under the plot of *K* = 7. (B) The NJ tree of the seven clusters for *K* = 7. Values shown next to each cluster number are *F*_ST_ values between each cluster and the common ancestral population.

The overall *F*_ST_ and *F’*_ST_ values were 0.581 and 0.739, respectively, indicating a high level of genetic differentiation among populations. This pattern was not changed when admixed individuals were removed from the data (*F*_ST_ = 0.521, *F’*_ST_ = 0.699). The average of the pairwise *F*_ST_ values among the M-type populations was higher than that among the S-type populations, and the difference was nearly significant (*P* = 0.067). Significant IBD was detected among the 7 populations, both with (*R*^2^ = 0.3447; *P* < 0.05) and without (*R*^2^ = 0.3435; *P* < 0.05) the admixed individuals included (Fig 2). In the STRUCTURE analysis, the mean probability of the data (LnP(D)) increased steadily up to *K* = 7 (S2 Fig) and *ΔK* suggested *K* = 7 as optimal (S2 Fig). At *K* = 2, ESAN 1 and 2 were grouped into cluster 1 and the remaining populations were assigned to cluster 2 (Fig 3). Thus, the clustering at *K* = 2 did not correspond to the M- and S-types. At *K* = 3, two M-type populations (KANT and SAND) in Wakayama Prefecture and one S-type population (SADO) were separated from IZU1 and IZU2. At *K* = 4, KANT was differentiated. At *K* = 5 and greater, five clusters corresponding to the five main sampling localities (Fig 1) were observed. At *K* = 6, SADO and SAND populations were shown to contain a considerable number of admixed individuals (with cluster 6). At *K* = 7, cluster 6 was further divided into two clusters and cluster 7 corresponded to the admixed cluster in SAND. The NJ tree for the seven clusters revealed two groups. In one group, cluster 1 (ESAN 1 and 2), 2 (IZU 1 and 2), 4 (KANT) and 5 (major cluster in SAND) were grouped together, while cluster 3 and 6 in SADO and cluster 7 for the admixed cluster in SAND were in the other group. The *F* values of each cluster (analogous to the *F*_ST_ values between each cluster and the assumed ancestral population) showed that clusters corresponding to M-type populations had larger values (0.627–0.751) than S-type populations (0.474–0.508, Fig 3).

### Inference of demographic history of each population

In DIYABC, the highest posterior probability was for scenario 1 (bottleneck model), and its 95% confidence interval (CI) did not overlap with those of the other two scenarios in each of the 7 populations, regardless of gametangia formation type (S5 Table). For scenario 1, the median values of the effective modern population size of *N*_1_ were well estimated in each population. The S-type populations had significantly larger *N*_1_ values (465–846) than the M-type ones (169–328; *t*-test, *P* < 0.05). However, the posterior distribution pattern suggested that other parameters were poorly estimated (S3 Fig and S6 Table), with the exception of the timing of the population size change event (*t*_1_) in the SADO population. In the SADO population, the median value of *t*_1_ was 2,940 generations ago (95% CI, 256–9,390), corresponding to 8,820 years ago (95% CI, 768–28,170). In all populations, all of the summary statistics showed no significant differences between the observed and simulated data, based on the posterior distributions (S6 Table), and the PCA showed that the observed data point was centered on the cluster of simulated data points, based on the posterior distributions (S4 Fig), suggesting that scenario 1 was a good fit to the observed data in all populations.

## Discussion

### Mating system differentiation, genetic diversity and effective population size

We found evidence for mixed mating in the northern type of diploid *C*. *falcatum*, as all but one population (SADO) had significantly positive *F*_IS_ values, ranging from 0.2 to 0.8, indicating it is highly likely that gametophytic selfing, sporophytic selfing, and outcrossing are all occurring in these populations. In contrast to our results, Chung et al. (2012) [47] examined sexual *C*. *falcatum* populations along the southern shores of South Korea, and showed that the *F*_IS_ values for these populations did not significantly deviate from zero, suggesting the prevalence of outcrossing. The *C*. *falcatum* individuals described in Chung et al. (2012) [47] may be the southern type, because Matsumoto (2003) [11] reported the distribution of the southern type in Nagasaki Pref., Japan (Fig 1), which is only 200 km away from southern South Korea, separated by the Tsushima Strait. Matsumoto (2003) [11] suggested that the southern type was an outcrosser based on its S-type of sexual expression of gametophytes, and the consistently low rates of sporophyte formation in isolated cultures. Because of the existence of these different mating systems, the two sexual diploid *C*. *falcatum* types provide an interesting experimental opportunity for future evolutionary studies pertaining to the transition between obligate outcrossing and mixed mating in homosporous ferns.

A cluster specific to the SADO and SAND populations, which are located far apart from one another, was detected in the STRUCTURE analysis (*K* = 6, cluster 6, Fig 3). One of the possible explanations for this cluster is long-distance dispersal of the southern type into the northern range followed by hybridization of the northern and southern types. Although we examined voucher specimens of SADO and SAND populations, there were no morphological differences between the pure and admixed individuals revealed in the STRUCTURE analysis. Additional studies including samples of the southern type are required to clarify the geographical distribution pattern of the two types of *C*. *falcatum*.

The *F*_IS_ values of M-type populations were significantly higher than those of S-type ones (Tables 1 and 2). Allelic richness (*A*_R_) and gene diversity (*h*) values of M-type populations were lower than those of S-type ones and the effective population size estimated by DIYABC also showed the same pattern (Tables 1 and 2). We acknowledge that there are several sources of uncertainty and assumptions in the ABC approach (e.g. assumptions about the generation time, overlapping generations, confidence intervals of the estimated parameters and the assumed model; for details see Tsuda et al. (2015) [48]). DIYABC does not assume gene flow after divergence, which may bias estimates of divergence time and effective population size when demographic scenarios for multiple samples (e.g. population, species) are examined [48,49]. For this reason, we did not include population splits in our scenarios and only inferred the temporal effective population size change in single populations. Moreover, the relatively high *F*’_ST_ value suggests that gene flow among populations might be restricted. Thus, although we need to consider the assumptions of the methods used in genetic analyses, we believe the results of ABC are informative regarding the differences between the two types of populations in our study. Indeed, although the inference of effective population size change in single populations is a simple ABC approach, Sakaguchi et al. (2013) [50] employed a similar approach to successfully detect geographic patterns in effective population size of conifer species in Australia.

To our knowledge, this is the first study to show that the type of gametangia formation of gametophytes affects the levels of inbreeding and genetic diversity in natural populations of homosporous ferns. It is well known that inbreeding species have lower neutral genetic diversity within populations compared to outcrossing taxa [51]. The correlation between genetic diversity and gametangia formation in M- and S-types of *C*. *falcatum* follows these general trends. The observed patterns could be due to several factors. Firstly, inbreeding is expected to reduce effective population size: *N*_e_ = *N* / (1+*F*_IS_) [52]. Secondly, recent empirical studies in seed plants have revealed that reduction of genetic diversity or effective population size are often greater than those expected from *F*_IS_ values alone [8], possibly because of linked selection owing to reduced recombination efficiency [53], and/or because of population bottlenecks. In the present study, despite their intermediate *F*_IS_, average gene diversity (*h*) of the M-type populations (0.152) was about half that of S-type populations (0.367), and the average *N*_e_ of the M-type populations (215) was about a third of that of S-type populations (675). Although the 95% CIs of the inferred *N*_e_ values in the ABC should be taken into consideration, these levels of reduction in *h* and *N*_e_ are comparable to the case of complete inbreeding, and seem to be greater than those expected from the intermediate *F*_IS_ values observed in M- and S-type populations (0.626 vs. 0.208). One possible explanation for population bottlenecks is colonization. The gametophytic selfing in ferns is hypothesized to be an advantage for long-distance colonization, as it enables a single spore to establish a new population [4,54,55]. For example, de Groot et al. (2012) [56] examined fern populations in a recently reclaimed Dutch polder land and concluded that the polder land was colonized via multiple independent single-spore colonization events in all four species studied. It is likely that simultaneous formation of both male and female gametangia and higher rates of selfing confer higher colonization ability to M-type individuals. This selfing ability is likely to be advantageous for range expansion and also following colonization. Both M- and S-type individuals of the northern type are lithophytes that grow on sea cliffs, a habitat that is vulnerable to disasters such as landslides. In fact, one population of the northern type in Fukushima Pref. was lost after the 2011 Great East Japan Earthquake and Tsunami [57]. DIYABC detected past population bottlenecks (Scenario 1, S1 Fig) in all populations regardless of M- or S-type (S4 Table), and this might reflect past episodes of local extinction and recolonization in unstable habitats. Although there is no apparent difference between the habitats of M- and S-types, it is possible that differences in magnitude or frequency of past colonization bottleneck events could result in significant differences in the *F*_ST_, genetic diversity and effective population sizes between M- and S-type populations.

### Population genetic structure

Previous studies using allozymes or microsatellites showed that the standardized *F’*_ST_ values are strongly variable in homosporous fern species (as they are in seed plant species), e.g. *F’*_ST_ = 0.068 for *Odontosoria chinensis* in Hawaii [58], *F’*_ST_ = 0.761 for *Dryopteris aemura* in Spain [59], *F’*_ST_ = 0.589 for *Cyrtomium falcatum* in Korea [47], and *F’*_ST_ = 0.414 for *Selliguea hastata* in Japan [60] (all *F’*_ST_ values calculated by R.I.). The *F*’_ST_ values (0.739) of the present study are relatively high. Interestingly, Korean outcrossing populations of *C*. *falcatum* [47] also have a relatively high overall *F’*_ST_ value (0.589) when compared to the northern type of *C*. *falcatum*, despite their putative outcrossing. This may indicate that this level of population differentiation is typical for the species and could be due to species dispersal ability and/or habitat preferences. For example, it is possible that a discontinuous geographical distribution of suitable habitat, such as crevices in sea cliffs or rocks near the seashore, is responsible for population differentiation. Significant IBD was detected in this study (Fig 2) and the STRUCTURE analysis did not show clear clustering corresponding to the M- and S-type populations (Fig 3). Although more populations would be required to conclude, we suggest that range-wide genetic structure of this species is not generated by M- and S-type differences, but rather by dispersal limitation. Significant IBD, related to past range shifts following climate change, has been detected in many plant species in the Japanese archipelago [61–63]. Significant IBD was also reported in a fern species, *Asplenium fontanum* subsp. *fontanum*, in Europe [64] and was likely caused by range expansion following the last glacial maximum (LGM). Similarly, past range shift in relation to the LGM was described in *Selliguea hastata* in Korea and Japan [60]. Although not well examined in palaeoecological studies of spore fossils, past distributional shifts in relation to the LGM might play an important role in generating the modern genetic structure of *C*. *falcatum*. Interestingly, although the time scale of population size change in the northern type of *C*. *falcatum* was well estimated in only one population (SADO, 8820 years ago, 95%CI: 1224–26610 years ago), this timing corresponds to a post-LGM recolonization scenario.

The M- and S-types of *C*. *falcatum* were significantly different in levels of selfing (*F*_IS_), genetic diversity, and effective population size. These results suggest that reproductive and demographic differences exist between the two types, despite the lack of genetic structure between them. The more severe population bottlenecks were inferred to have occurred in the M-type populations rather than the S-types. This implies that local extinction and recolonization events could provide opportunities for the maintenance of the M-type populations, and possibly for increasing the frequency of the M-type individual in some cases, even under occasional immigration of the S-type individuals. Evolution of selfing via transmission advantage operates only under a low genetic load, and fixed selfing is expected [7]. Therefore, the existence of mixed mating even in the M-type populations would suggest that reproductive assurance, rather than transmission advantage, is the main factor affecting the evolution of selfing in this species. If this is the case, the M-type would be advantageous in small and disturbed populations, while the S-type would be advantageous in large and stable populations.

In this study, we discuss results obtained after removing putative admixed individuals in SADO and SAND populations. Subpopulation genetic structure within these two populations may be one of the reasons why a clearer difference in genetic diversity between the S- and M-types was detected only after the removing admixed samples. However, it is difficult to discuss the origin of cluster 6 and 7 in detail with the current dataset. One possible explanation is migration and hybridization of northern and southern types of *C*. *falcatum* in these areas, although this was not expected given the morphological observations of Matsumoto 2003 [11]. However, our on-going study based on restriction site associated DNA sequencing (RAD-seq) supports this hypothesis (Imai et al. unpublished). We would need to sample individuals covering the species entire distributional range, including both southern and northern types, and evaluate genetic variation using several types of markers to clarify sympatric distribution and hybridization.

## Supporting Information

S1 Fig Demographic models used in DIYABC for each population of *C*. *falcatum*. Scenario 1. Bottleneck model: the ancestral effective population size (*N*_a_) was changed at *t*_1_ to the modern effective population size (*N*_1_) and *N*_1_ was set to be smaller than *N*_a_ Scenario 2. Constant model: the ancestral effective population size (*N*_b_) and the modern one (*N*_1_) were set to be equal, assuming the effective population size has not changed. Scenario 3. Expansion model: the ancestral effective population size (*N*_c_) was changed at *t*_1_ to the modern effective population size (*N*_1_) and *N*_1_ was set to be larger than *N*_c_.
(EPS)

S2 Fig The values of posterior probability of the data (Ln P(D)) from 10 runs for each value of *K* (1–15; A) and *ΔK* (right B). (EPS)

S3 Fig Prior and posterior distributions for each parameter obtained by DIYABC analysis for each population. X axis indicates values for the parameter described in the title of each graph; pmic: the parameter of the geometric distribution to generate multiple stepwise mutations; smic: Mean mutation rate of single nucleotide indel; μmic: mean mutation rate of SSR. Y axis indicates probability of prior and posterior.
(PPTX)

S4 Fig Principal Component Analysis (PCA) score plot obtained from DIYABC analysis for each population. PCA plots of prior, posterior and observed data set for summary statistics.
(PPTX)

S1 Table Primer sequences, repeat motifs, and accession numbers of source sequences for eight microsatellite markers developed in this study. (DOCX)

S2 Table Genetic diversity indices and inbreeding coefficient values for seven populations of diploid *Cyrtomium falcatum*. (DOCX)

S3 Table Null allele frequencies at each locus estimated by INEST2. (DOC)

S4 Table Inbreeding coefficient (*F*_IS_) values estimated by INEST2 for seven populations of the northern type of diploid *Cyrtomium falcatum*. (DOCX)

S5 Table Posterior probability of each scenario by DIYABC. (XLSX)

S6 Table Demographic parameters of scenario 1 obtained by DIYABC. (XLSX)

S7 Table Genotype list of all samples. (XLSX)
