# Supplementary material for: The Relationship between Mating System and Genetic Diversity in Diploid Sexual Populations of Cyrtomium falcatum in Japan
Source: PLoS One. 2016 Oct 5;11(10):e0163683. doi: 10.1371/journal.pone.0163683 (PMC5051678; doi:10.1371/journal.pone.0163683)
Supplement: S3 Fig — X axis indicates values for the parameter described in the title of each graph; pmic: the parameter of the geometric distribution to generate multiple stepwise mutations; smic: Mean mutation rate of single nucleotide indel; μmic: mean mutation rate of SSR. Y axis indicates probability of prior and posterior. (PPTX) [file pone.0163683.s003.pptx]

## Slide 1
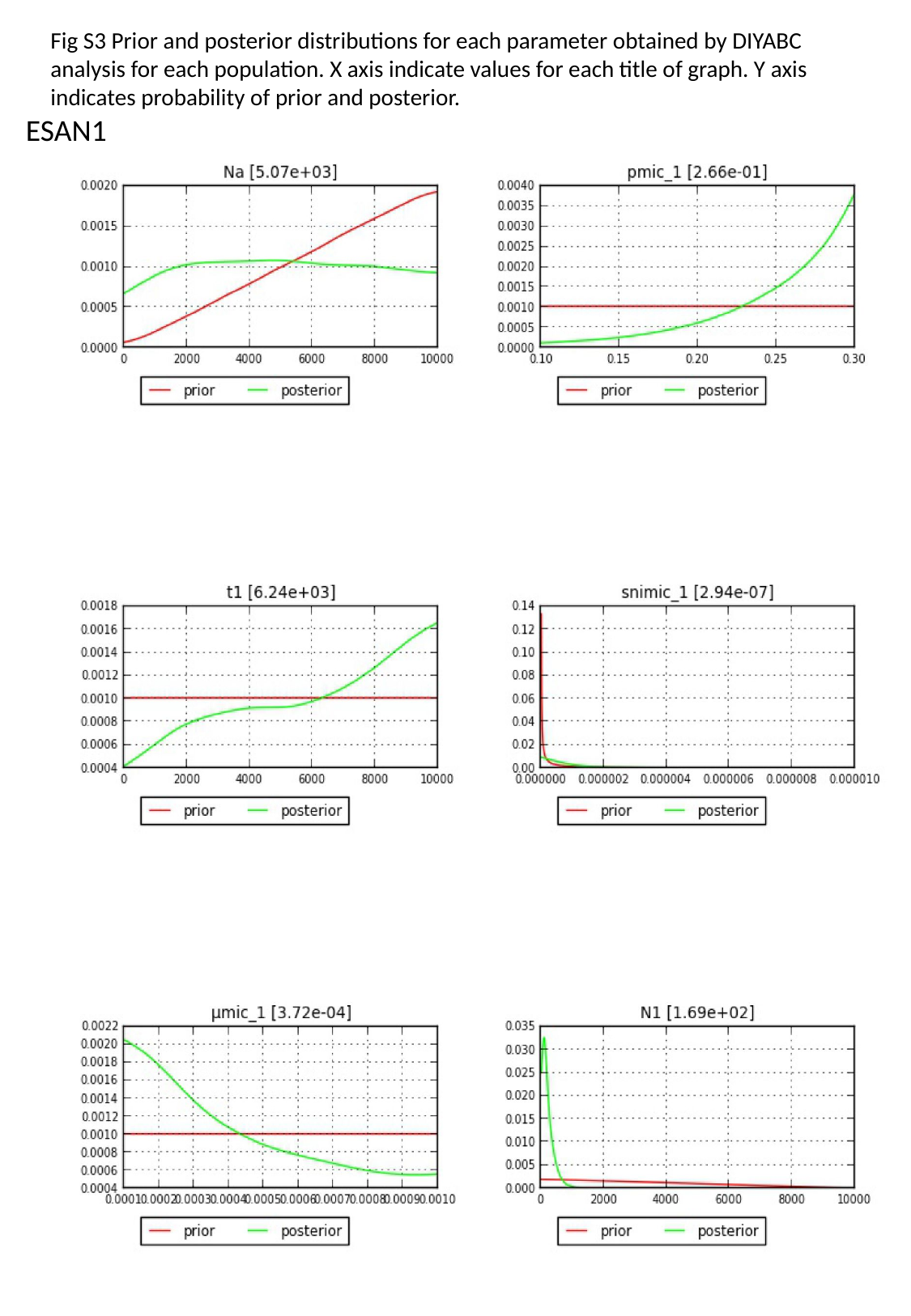

Fig S3 Prior and posterior distributions for each parameter obtained by DIYABC analysis for each population. X axis indicate values for each title of graph. Y axis indicates probability of prior and posterior.
ESAN1

## Slide 2
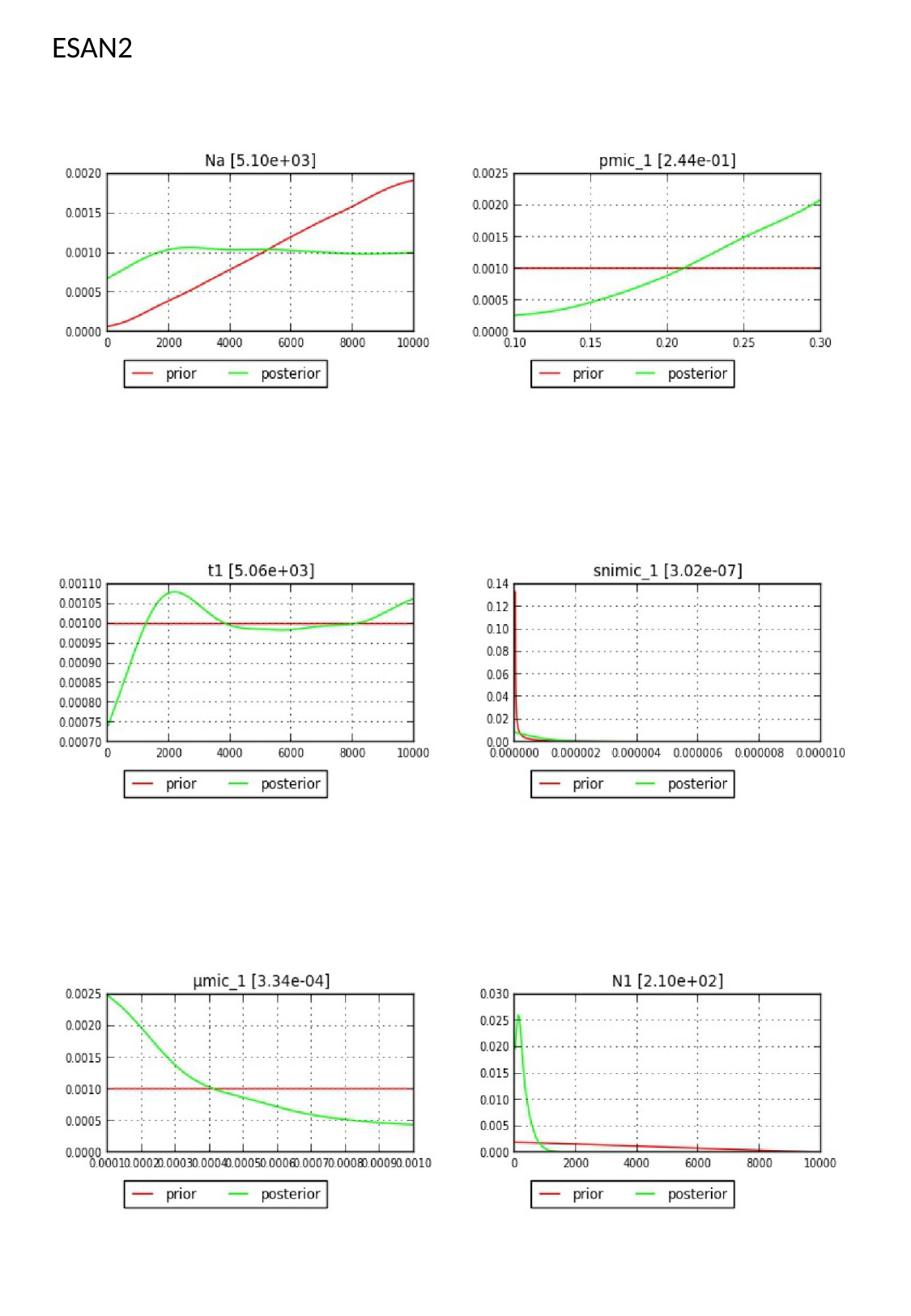

ESAN2

## Slide 3
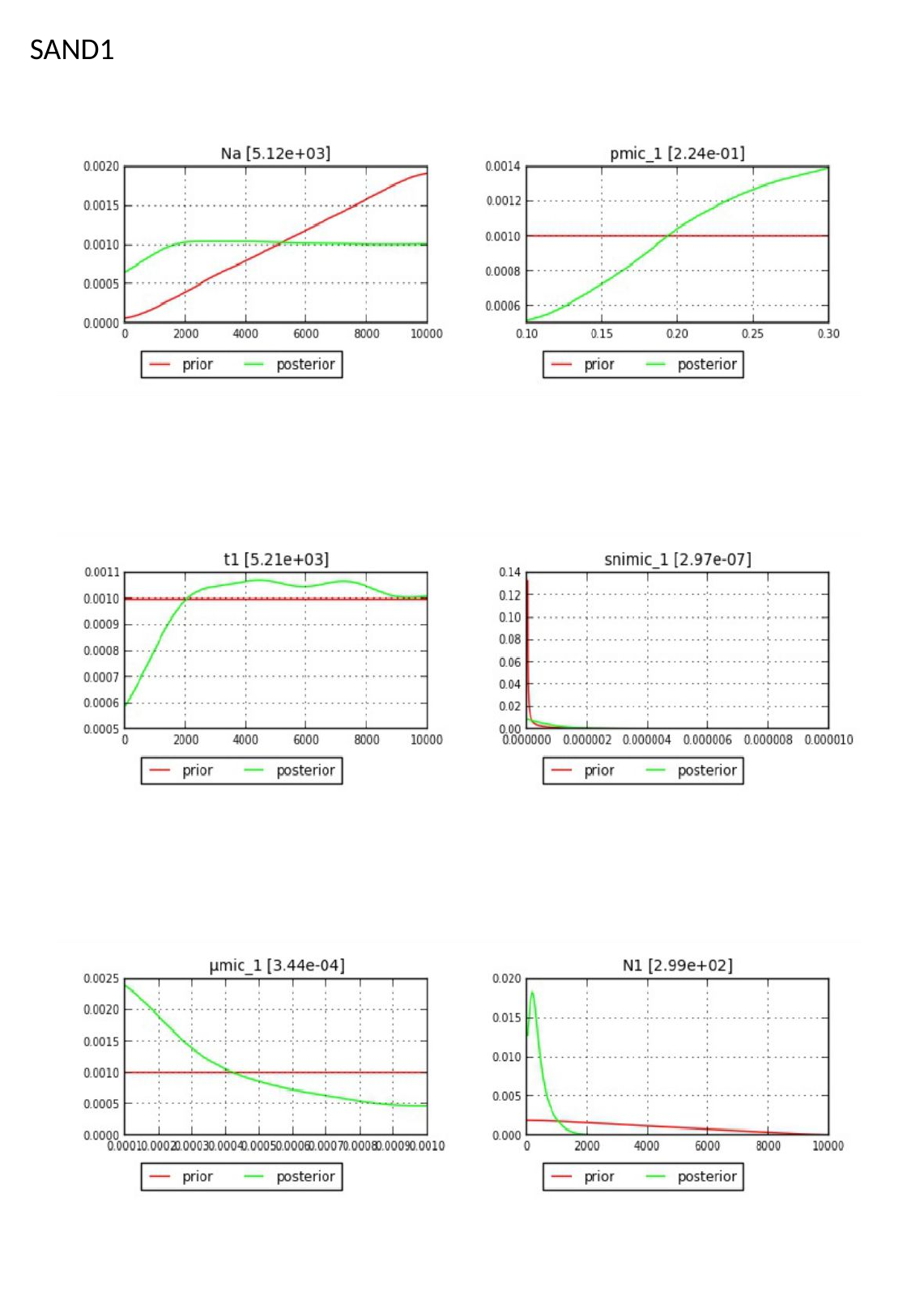

SAND1

## Slide 4
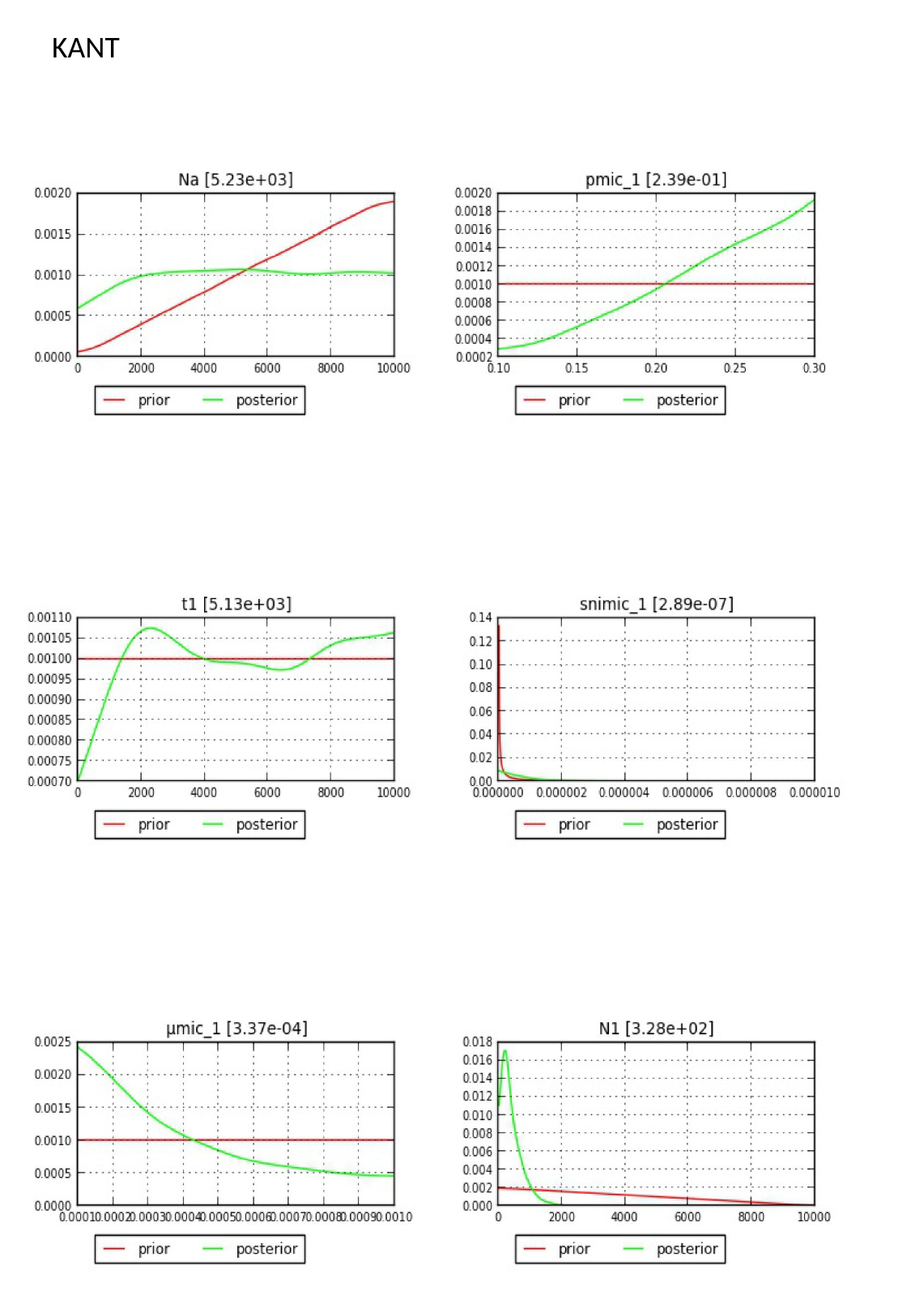

KANT

## Slide 5
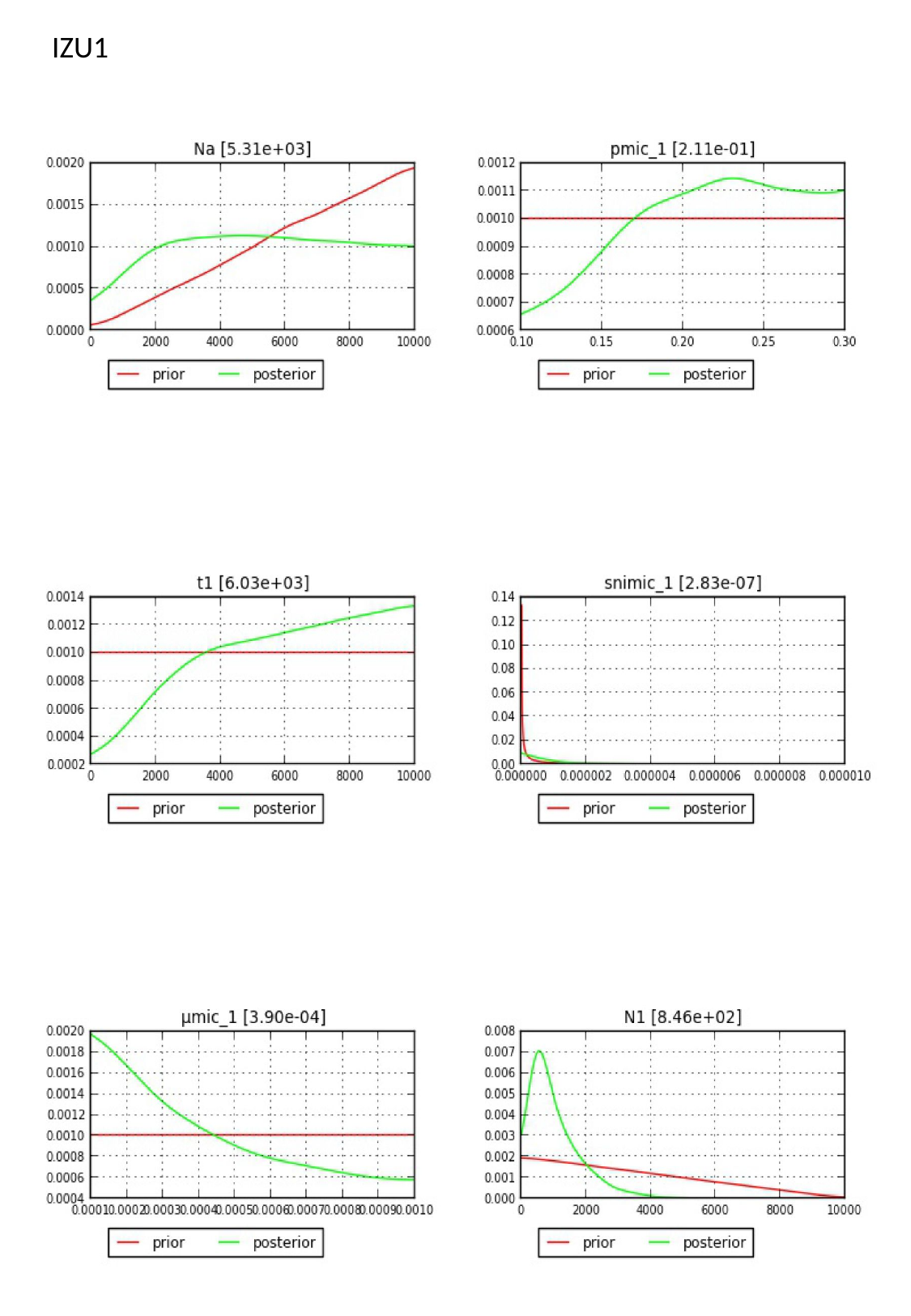

IZU1

## Slide 6
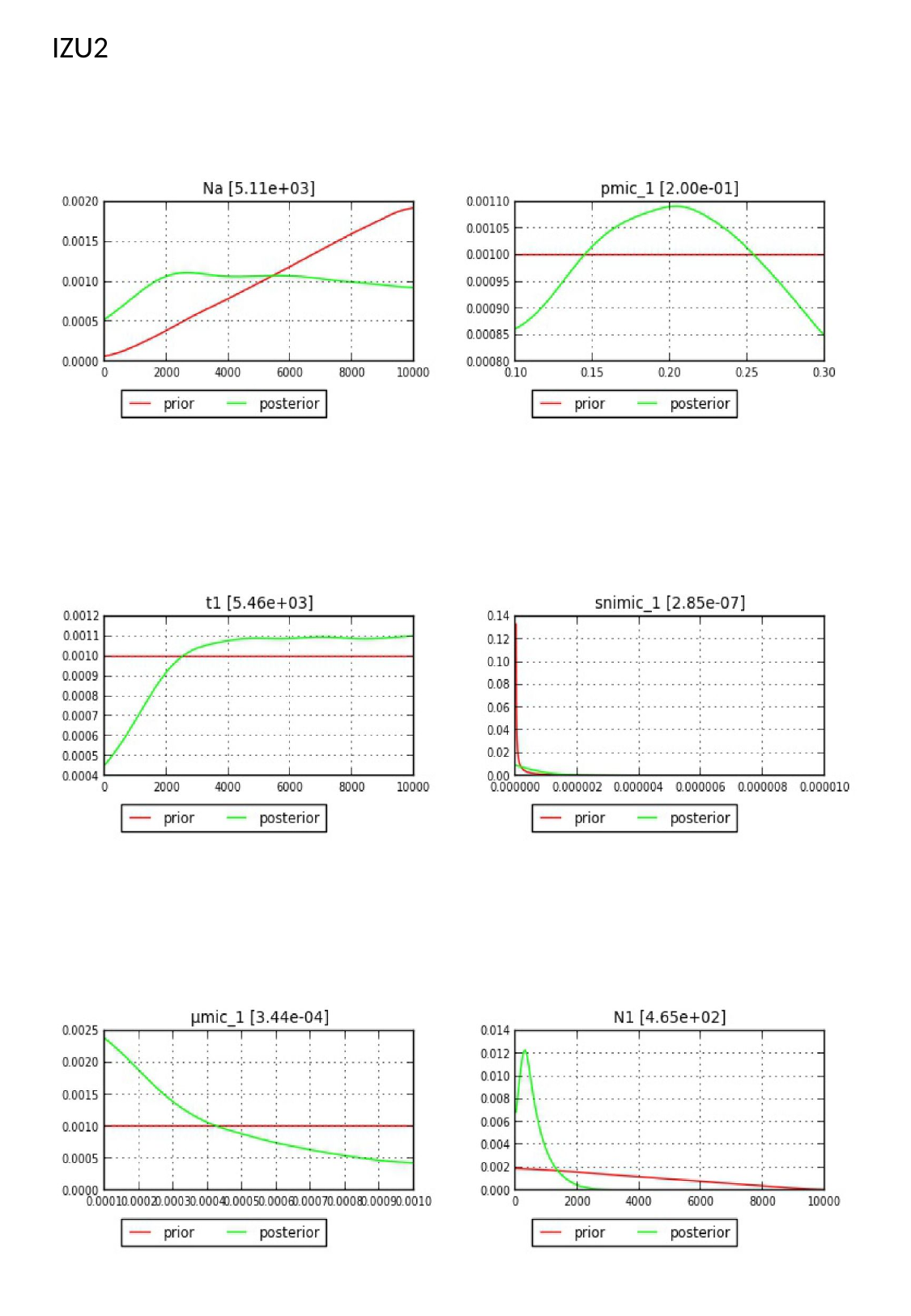

IZU2

## Slide 7
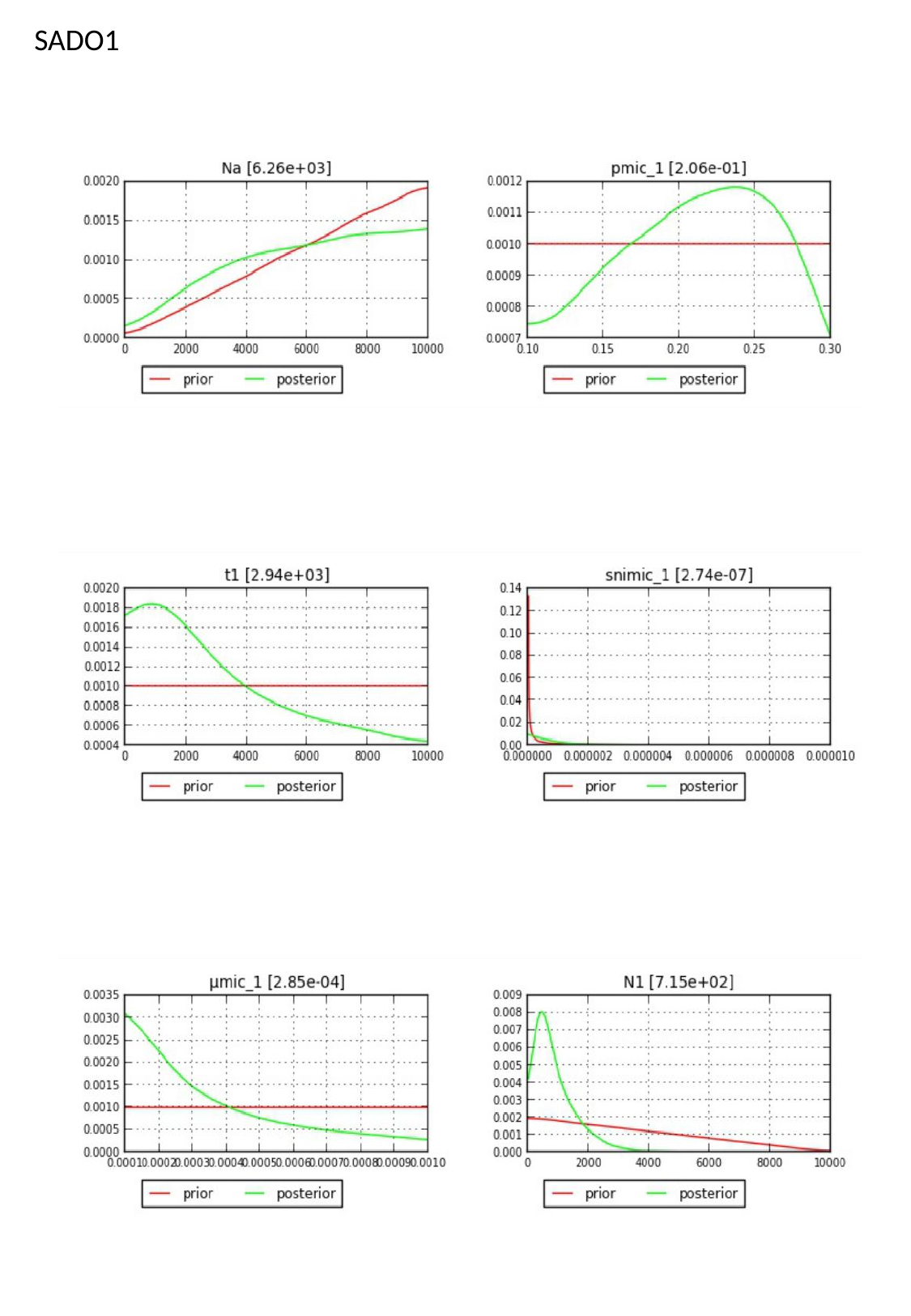

SADO1
