# Supplementary material for: The Relationship between Mating System and Genetic Diversity in Diploid Sexual Populations of Cyrtomium falcatum in Japan
Source: PLoS One. 2016 Oct 5;11(10):e0163683. doi: 10.1371/journal.pone.0163683 (PMC5051678; doi:10.1371/journal.pone.0163683)
Supplement: S4 Fig — PCA plots of prior, posterior and observed data set for summary statistics. (PPTX) [file pone.0163683.s004.pptx]

## Slide 1
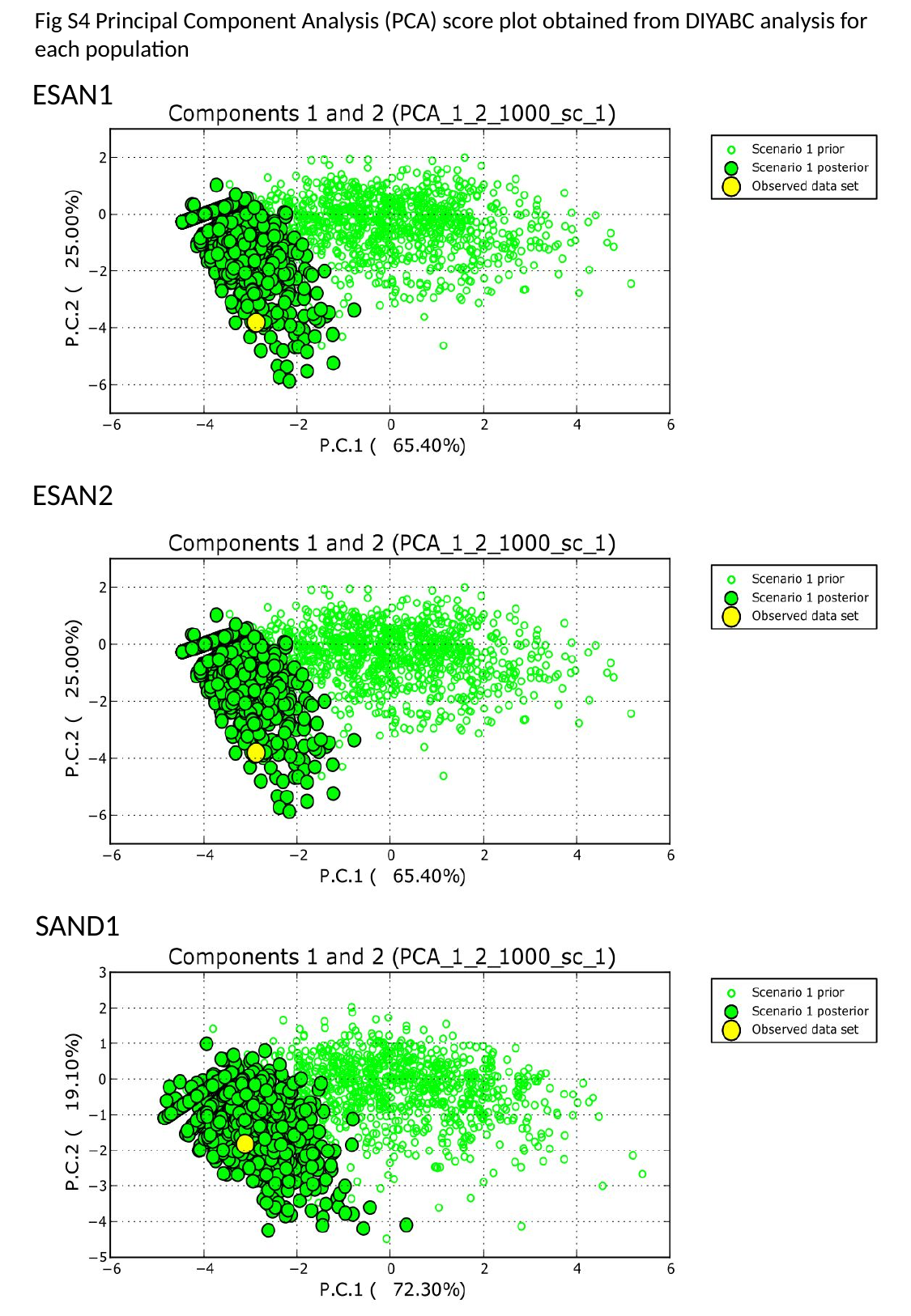

Fig S4 Principal Component Analysis (PCA) score plot obtained from DIYABC analysis for each population
ESAN1
ESAN2
SAND1

## Slide 2
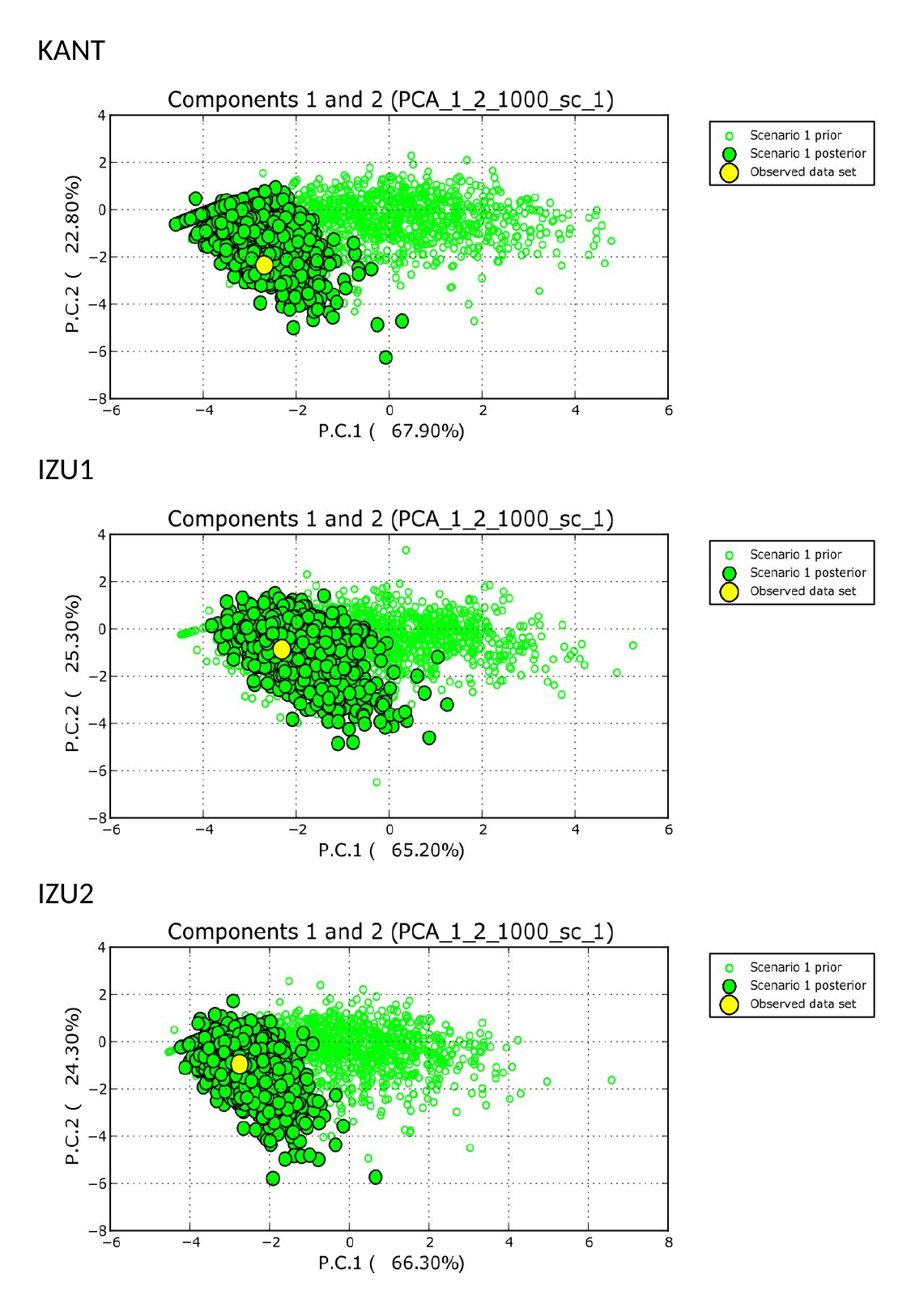

KANT
IZU1
IZU2

## Slide 3
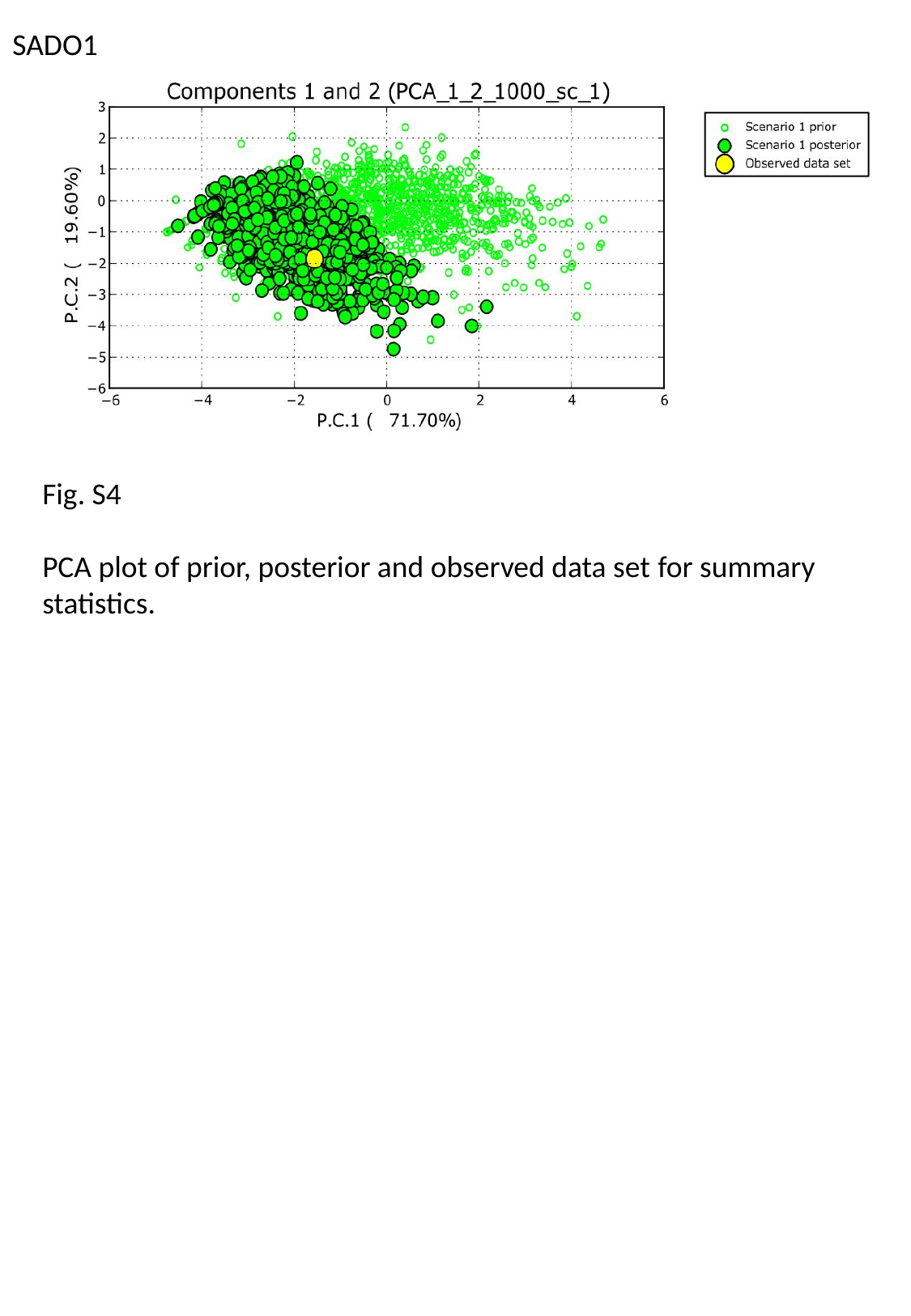

SADO1
Fig. S4
PCA plot of prior, posterior and observed data set for summary statistics.
