# Supplementary material for: The Relationship between Mating System and Genetic Diversity in Diploid Sexual Populations of Cyrtomium falcatum in Japan
Source: PLoS One. 2016 Oct 5;11(10):e0163683. doi: 10.1371/journal.pone.0163683 (PMC5051678; doi:10.1371/journal.pone.0163683)
Supplement: S1 Table — (DOCX) [file pone.0163683.s005.docx]

| Table S1. Primer sequences, repeat motifs, and accession numbers of source sequences for eight microsatellite markers developed in this study. | | | |
| --- | --- | --- | --- |
| Locus | Primer sequence (5′–3′) | Repeat motif | Accession number |
| CFL-079* | F: pig1-ACGAAGAAGGACGAGTAGC | (AC)_6_(AG)_10_ | LC055975 |
| CFL-079* | R: ACACACACACAGAGAGAGAGAG | (AC)_6_(AG)_10_ | LC055975 |
| CFL-C32* | F: CTCATGGGACTTTTTGTGTC | (AC)_6_(AG)_10_ | LC055976 |
| CFL-C32* | R: ACACACACACAGAGAGAGAGAG | (AC)_6_(AG)_10_ | LC055976 |
| CFL-Z03# | F: u19-AATGGAAGAGGGCACGAGTA | (GA)_8_ | LC055977 |
| CFL-Z03# | R: pig2-GCATGTCCAAAGGAGTGACTT | (GA)_8_ | LC055977 |
| CFL-B02# | F: u19-GCTTGCTTGACAGAGACACG | (GA)_14_, A_7_ | LC055978 |
| CFL-B02# | R: pig2-TATGAACGGATAGTGCCACG | (GA)_14_, A_7_ | LC055978 |
| CFL-B12# | F: u19-CCGTTGAAGGTGGGAAGTAA | (TC)_12_ | LC055979 |
| CFL-B12# | R: pig2-AGCCTCCATGCCTCCTTTAT | (TC)_12_ | LC055979 |
| CFL-B13# | F: u19-TCGGCTCTACCTCCTCTCAA | (AC)_11_ | LC055980 |
| CFL-B13# | R: pig2-ATGAGTGCATATGGGCAACA | (AC)_11_ | LC055980 |
| CFL-B16# | F: u19-GTAAGTGGGCACTTTCCCTG | (GT)_11_ | LC055981 |
| CFL-B16# | R: pig2-GCGCTAAGGTTGTTCGTCTC | (GT)_11_ | LC055981 |
| CFL-B17# | F: u19-GACGAGGGCGTAAATGAGAA | (TC)_5_, (AC)_11_, A_4_ | LC055982 |
| CFL-B17# | R: pig2-GCCATAACGTCAAGGCAAGT | (TC)_5_, (AC)_11_, A_4_ | LC055982 |
|  |  |  |  |
| *, markers developed by the method of Lian *et al*. (2006); #, markers developed by next generation sequencing. | | | |
| u19, 5′-GGTTTTCCCAGTCACGACG-3′; pig1, 5′-GTTTCTT-3′; pig2, 5′-GTTT-3′. | | | |
