# Supplementary material for: The Relationship between Mating System and Genetic Diversity in Diploid Sexual Populations of Cyrtomium falcatum in Japan
Source: PLoS One. 2016 Oct 5;11(10):e0163683. doi: 10.1371/journal.pone.0163683 (PMC5051678; doi:10.1371/journal.pone.0163683)
Supplement: S2 Table — (DOCX) [file pone.0163683.s006.docx]

Table S2. Genetic diversity indices and inbreeding coefficient values for seven populations of diploid *Cyrtomium falcatum*.

| Locus | *N*_A_ | Size range (bp) |  | M_type populations | | | | |  | S_type populations | | | |
| --- | --- | --- | --- | --- | --- | --- | --- | --- | --- | --- | --- | --- | --- |
|  |  |  |  | ESAN1 | ESAN2 | SAND  (SAND#) | KANT | Mean |  | IZU1 | IZU2 | SADO  (SADO# | Mean |
|  |  |  |  | *n*=41 | *n*=36 | *n*=21  (n=16) | *n*=28 |  |  | *n*=42 | *n*=35 | *n*=30  (n=17) |  |
| CFL-079 | 14 | 316-340 | *N*_A_ | 3 | 2 | 6  (3) | 5 | 4.000 |  | 5 | 3 | 4  (2) | 4.000 |
|  |  |  | *A*_R_ | 2.075 | 1.754 | 5.642  (1.50) | 4.536 | 3.502 |  | 3.698 | 3.000 | 3.850  (1.993) | 3.516 |
|  |  |  | *H*_E_ | 0.073 | 0.056 | 0.364  (0.118) | 0.417 | 0.228 |  | 0.568 | 0.589 | 0.600  (0.484) | 0.586 |
|  |  |  | *F*_IS_ | 0.664 | **1.000** | **0.451**  (0.500) | **0.556** | 0.668 |  | 0.142 | **0.382** | -0.353  (-0.684) | 0.171 |
| CFL-C32 | 9 | 181-202 | *N*_A_ | 3 | 3 | 5  (3) | 2 | 3.25 |  | 8 | 6 | 5  (2) | 6.333 |
|  |  |  | *A*_R_ | 2.352 | 2.566 | 4.681  (1.5) | 2.000 | 2.900 |  | 6.623 | 5.883 | 4.497  (1.250) | 5.668 |
|  |  |  | *H*_E_ | 0.139 | 0.292 | 0.361  (0.118) | 0.300 | 0.273 |  | 0.821 | 0.809 | 0.599  (0.0601) | 0.743 |
|  |  |  | *F*_IS_ | 0.123 | **0.886** | 0.584  (0.500) | **1.000** | 0.648 |  | **0.258** | 0.143 | **0.236**  **(0.000)** | 0.212 |
| CFL-Z03 | 7 | 227-253 | *N*_A_ | 3 | 1 | 1  (1) | 1 | 1.500 |  | 3 | 2 | 6  (6) | 3.667 |
|  |  |  | *A*_R_ | 2.450 | 1.000 | 1.000  (1.000) | 1.000 | 1.363 |  | 2.649 | 2.000 | 5.005  (3.113) | 3.218 |
|  |  |  | *H*_E_ | 0.109 | 0.000 | 0.000  (0.000) | 0.000 | 0.027 |  | 0.498 | 0.508 | 0.701  (0.626) | 0.569 |
|  |  |  | *F*_IS_ | 0.489 | - | -  (-) | - | 0.489 |  | **0.618** | 0.262 | 0.213  (-0.480) | 0.364 |
| CFL-B02 | 14 | 130-155 | *N*_A_ | 1 | 4 | 5  (3) | 1 | 2.75 |  | 4 | 2 | 8  (5) | 4.67 |
|  |  |  | *A*_R_ | 1.000 | 3.400 | 4.990  (2.180) | 1.000 | 2.598 |  | 3.108 | 1.754 | 6.889  (3.476) | 3.917 |
|  |  |  | *H*_E_ | 0.000 | 0.444 | 0.664  (0.420) | 0.000 | 0.277 |  | 0.184 | 0.059 | 0.831  (0.706) | 0.358 |
|  |  |  | *F*_IS_ | - | **0.937** | **0.570**  (**0.860**) | - | 0.754 |  | 0.207 | 1.000 | -0.025  (-0.384) | 0.394 |
| CFL-B12 | 5 | 172-184 | *N*_A_ | 3 | 2 | 4  (4) | 3 | 3.00 |  | 2 | 1 | 3  (3) | 2.00 |
|  |  |  | *A*_R_ | 2.712 | 1.725 | 4.000  (2.632) | 2.607 | 2.761 |  | 1.794 | 1.000 | 3.000  (2.889) | 1.931 |
|  |  |  | *H*_E_ | 0.167 | 0.056 | 0.526  (0.435) | 0.476 | 0.306 |  | 0.070 | 0.000 | 0.654  (0.650) | 0.241 |
|  |  |  | *F*_IS_ | **0.550** | 1.000 | **0.888**  (**0.836**) | **0.850** | 0.822 |  | 0.661 | - | **0.631**  (0.656) | 0.646 |
| CFL-B13 | 8 | 237-251 | *N*_A_ | 1 | 1 | 3  (3) | 3 | 2.00 |  | 3 | 4 | 2  (2) | 3.00 |
|  |  |  | *A*_R_ | 1.000 | 1.000 | 3.000  (2.819) | 2.996 | 1.999 |  | 2.434 | 3.217 | 2.000  (1.878) | 2.550 |
|  |  |  | *H*_E_ | 0.000 | 0.000 | 0.663  (0.605) | 0.389 | 0.263 |  | 0.252 | 0.166 | 0.384  (0.327) | 0.267 |
|  |  |  | *F*_IS_ | - | - | **0.774**  (**0.793**) | 0.111 | 0.443 |  | **0.898** | **0.655** | 0.392  (0.484) | 0.648 |
| CFL-B16 | 2 | 187-189 | *N*_A_ | 2 | 2 | 2  (2) | 1 | 1.75 |  | 2 | 2 | 2  (2) | 2.00 |
|  |  |  | *A*_R_ | 1.937 | 1.977 | 1.999  (1.444) | 1.000 | 1.728 |  | 2.000 | 2.000 | 1.969  (1.566) | 1.990 |
|  |  |  | *H*_E_ | 0.117 | 0.143 | 0.179  (0.116) | 0.000 | 0.110 |  | 0.471 | 0.321 | 0.126  (0.161) | 0.306 |
|  |  |  | *F*_IS_ | **0.792** | 0.364 | 0.467  (1.000) | - | 0.541 |  | 0.276 | -0.038 | -0.055  (-0.067) | 0.061 |
| CFL-B17 | 5 | 275-289 | *N*_A_ | 1 | 2 | 4  (1) | 2 | 2.25 |  | 3 | 2 | 3  (2) | 2.67 |
|  |  |  | *A*_R_ | 1.000 | 2.000 | 3.698  (1.000) | 1.992 | 2.173 |  | 2.380 | 1.985 | 2.998  (1.989) | 2.454 |
|  |  |  | *H*_E_ | 0.000 | 0.275 | 0.233  (0.000) | 0.150 | 0.165 |  | 0.160 | 0.159 | 0.604  (0.472) | 0.308 |
|  |  |  | *F*_IS_ | - | 0.039 | -0.073  (-) | -0.067 | -0.034 |  | -0.069 | -0.079 | 0.117  (-0.091) | -0.010 |
| Mean | 8 | - | *N*_A_ | 2.13 | 2.125 | 3.75  (2.5) | 2.25 | 2.56 |  | 3.75 | 2.75 | 4.125  (3) | 3.541 |
|  |  |  | *A*_R_ | 1.816 | 1.928 | 3.626  (1.759) | 2.141 | 2.378 |  | 3.086 | 2.467 | 3.776 | 3.110 |
|  |  |  | *H*_E_ | 0.076 | 0.158 | 0.374  0.227 | 0.217 | 0.206 |  | 0.378 | 0.326 | 0.562 | 0.422 |
|  |  |  | *F*_IS_^*^ | **0.501** | **0.671** | **0.602**  (**0.794**) | **0.560** | 0.5835 |  | **0.340** | **0.220** | **0.157**  (-0.100) | 0.239 |

Bold type indicates significant *F*_IS_ values (p<0.00089, simple Bonferroni correction for 5% level); *, multi locus estimate.

*A*_R_, Allelic richness, and *H*_E_, Expected heterozygosity, are indexes of genetic diversity. The *F*_IS_ is Wright's fixation index, that index reflects selfing rate.

#: Excluding subpopulation detected by STRUCTURE
