# Supplementary material for: The Relationship between Mating System and Genetic Diversity in Diploid Sexual Populations of Cyrtomium falcatum in Japan
Source: PLoS One. 2016 Oct 5;11(10):e0163683. doi: 10.1371/journal.pone.0163683 (PMC5051678; doi:10.1371/journal.pone.0163683)
Supplement: S3 Table — (DOC) [file pone.0163683.s007.doc]

Table S3. Null allele frequencies at each locus estimated by INEST2

| Locus |  | M_type population | | | | |  | S-type population | | | |
| --- | --- | --- | --- | --- | --- | --- | --- | --- | --- | --- | --- |
|  |  | ESAN1 | ESAN2 | SAND  (SAND1 ) | KANT | Average |  | IZU1 | IZU2 | SADO  (SADO1 ) | Average |
|  |  | n=42 | n=36 | n=21  (n=16) | n=28 |  |  | n=42 | n=35 | n=30  (n=17) |  |
| CFL-079 | Freq. of null alleles | 0.00835 | 0.0668 | 0.0398  (0.014) | 0.0572 | 0.0431 |  | 0.0653 | 0.0136 | 0.00940  (0.00633) | 0.0324 |
|  | Low (95%) | 0.000 | 0.000 | 0.000  (0.000) | 0.000 | 0.000 |  | 0.000 | 0.000 | 0.000  (0.000) | 0.000 |
|  | High (95%) | 0.0475 | 0.179 | 0.139  (0.0726) | 0.189 | 0.139 |  | 0.176 | 0.0835 | 0.0450  (0.036) | 0.114 |
|  |  |  |  |  |  |  |  |  |  |  |  |
| CFL-C32 | Freq. of null alleles | 0.00653 | 0.184 | 0.0486  (0.015) | 0.253 | 0.120 |  | 0.0333 | 0.00813 | 0.0661  (0.0239) | 0.0502 |
|  | Low (95%) | 0.000 | 0.000 | 0.000  (0.000) | 0.000 | 0.000 |  | 0.000 | 0.000 | 0.000  (0.000) | 0.000 |
|  | High (95%) | 0.0378 | 0.350 | 0.167  (0.0757) | 0.466 | 0.253 |  | 0.0979 | 0.0480 | 0.247  (0.1416) | 0.135 |
|  |  |  |  |  |  |  |  |  |  |  |  |
| CFL-Z03 | Freq. of null alleles | 0.133 | 0.171 | 0.144  (0.1141) | 0.199 | 0.161 |  | 0.0518 | 0.0524 | 0.0747  (0.00616) | 0.0525 |
|  | Low (95%) | 0.000 | 0.000 | 0.000  (0.000) | 0.000 | 0.000 |  | 0.000 | 0.000 | 0.000  (0.000) | 0.000 |
|  | High (95%) | 0.3054 | 0.345 | 0.359  (0.3107) | 0.418 | 0.356 |  | 0.176 | 0.217 | 0.195  (0.0365) | 0.183 |
|  |  |  |  |  |  |  |  |  |  |  |  |
| CFL-B02 | Freq. of null allele | 0.00738 | 0.0168 | 0.0181  (0.0179) | 0.0200 | 0.0150 |  | 0.0563 | 0.0573 | 0.0114  (0.00718) | 0.0343 |
|  | Low (95%) | 0.000 | 0.000 | 0.000  (0.000) | 0.000 | 0.000 |  | 0.000 | 0.000 | 0.000  (0.000) | 0.000 |
|  | High (95%) | 0.0428 | 0.0825 | 0.0782  (0.0914) | 0.0986 | 0.0728 |  | 0.154 | 0.245 | 0.0522  (0.0382 ) | 0.124 |
|  |  |  |  |  |  |  |  |  |  |  |  |
| CFL-B12 | Freq. of null alleles | 0.0403 | 0.0123 | 0.212  (0.1338) | 0.0403 | 0.0760 |  | 0.0310 | 0.0114 | 0.246  (0.1931 ) | 0.0894 |
|  | Low (95%) | 0.000 | 0.000 | 0.000  (0.000) | 0.000 | 0.000 |  | 0.000 | 0.000 | 0.0977  (0.000) | 0.0252 |
|  | High (95%) | 0.138 | 0.0610 | 0.395  (0.3185) | 0.186 | 0.194 |  | 0.121 | 0.0675 | 0.400  (0.3851 ) | 0.190 |
|  |  |  |  |  |  |  |  |  |  |  |  |
| CFL-B13 | Freq. of null alleles | 0.00734 | 0.0107 | 0.0760  (0.0567) | 0.0526 | 0.0366 |  | 0.209 | 0.0200 | 0.0671  (0.0468 ) | 0.0949 |
|  | Low (95%) | 0.000 | 0.000 | 0.000  (0.000) | 0.000 | 0.000 |  | 0.000 | 0.000 | 0.000  (0.000) | 0.000 |
|  | High (95%) | 0.0362 | 0.0527 | 0.212  (0.182) | 0.174 | 0.118 |  | 0.350 | 0.113 | 0.207  (0.208 ) | 0.213 |
|  |  |  |  |  |  |  |  |  |  |  |  |
| CFL-B16 | Freq. of null alleles | 0.0100 | 0.0762 | 0.0210  (0.0173) | 0.019 | 0.0314 |  | 0.0523 | 0.0318 | 0.0285  (0.0172 ) | 0.0372 |
|  | Low (95%) | 0.000 | 0.000 | 0.000  (0.000) | 0.000 | 0.000 |  | 0.000 | 0.000 | 0.000  (0.000 ) | 0.000 |
|  | High (95%) | 0.0586 | 0.204 | 0.0926  (0.0936) | 0.0966 | 0.111 |  | 0.148 | 0.142 | 0.124  (0.098 ) | 0.135 |
|  |  |  |  |  |  |  |  |  |  |  |  |
| CFL-B17 | Freq. of null alleles | 0.0915 | 0.0408 | 0.0426  (0.0669) | 0.0933 | 0.0673 |  | 0.0472 | 0.00758 | 0.0319  (0.0125 ) | 0.0269 |
|  | Low (95%) | 0.000 | 0.000 | 0.000  (0.000) | 0.000 | 0.000 |  | 0.000 | 0.000 | 0.000  (0.000 ) | 0.000 |
|  | High (95%) | 0.244 | 0.125 | 0.131  (0.2102) | 0.263 | 0.190 |  | 0.142 | 0.0469 | 0.123  (0.0735 ) | 0.097 |
