# Supplementary material for: The Relationship between Mating System and Genetic Diversity in Diploid Sexual Populations of Cyrtomium falcatum in Japan
Source: PLoS One. 2016 Oct 5;11(10):e0163683. doi: 10.1371/journal.pone.0163683 (PMC5051678; doi:10.1371/journal.pone.0163683)
Supplement: S4 Table — (DOCX) [file pone.0163683.s008.docx]

| Table S4. Inbreeding coefficient (*F*_IS_) values estimated by INEST2 for seven populations of northern type of diploid *Cyrtomium falcatum*. | | | | | | | | | | |
| --- | --- | --- | --- | --- | --- | --- | --- | --- | --- | --- |
|  | M_type population | | | | | S_type population | | | |  |
|  | ESAN1 | ESAN2 | SAND | KANT | Mean | IZU1 | IZU2 | SADO | Mean |  |
|  | *n*=41 | *n*=36 | *n*=16 | *n*=28 |  | *n*=42 | *n*=35 | *n=*17 |  |  |
|  |  |  | (n=21) |  |  |  |  | (n=30) |  |  |
| *F*_IS_ mean | 0.484 | 0.634 | 0.772 | 0.478 | 0.592 | 0.274 | 0.225 | 0.0197 | 0.173 |  |
|  |  |  | (0.635) |  | (0.558) |  |  | (0.033) | (0.177) |  |
| Low (95%) | 0.205 | 0.435 | 0.567 | 0.211 | 0.355 | 0.140 | 0.210 | 0.000 | 0.117 |  |
|  |  |  | (0.504) |  | (0.339) |  |  | (0.000) | (0.117) |  |
| High (95%) | 0.720 | 0.815 | 0.942 | 0.699 | 0.794 | 0.408 | 0.372 | 0.0672 | 0.282 |  |
|  |  |  | (0.759) |  | (0.748) |  |  | (0.0962) | (0.292) |  |
| *F*_IS_ (Fixation index) estimated by INEST2. INEST2 estimates FIS with correcting effect of null alleles. | | | | | | | | | | |
